# Allicin disrupts cardiac Cav1.2 channels via trafficking

**DOI:** 10.1080/13880209.2019.1577469

**Published:** 2019-03-30

**Authors:** Dan Han, Lingping Xu, Peng Liu, Yingying Liu, Chaofeng Sun, Yanrong Yin

**Affiliations:** a Arrhythmia Unit, Department of Cardiology, The First Affiliated Hospital of Xi’an Jiaotong University, Xi’an, China;; b Xianyang Central Hospital, Xianyang, China

**Keywords:** Antiarrhythmia, trafficking dysfunction

## Abstract

**Context:** Allicin is a potential antiarrhythmic agent. The antiarrhythmic properties of allicin rely on its blockade of various cardiac ion channels. The l-type calcium (Cav1.2) channel provides a pivotal substrate for cardiac electrophysiologic activities. The mechanism of allicin on Cav1.2 remains unclear.

**Objective:** This study evaluated the potential of allicin on the synthesis and trafficking of Cav1.2 channels.

**Materials and methods:** Primary cardiomyocytes (CMs) from neonatal Sprague-Dawley (SD) rats were exposed to allicin (0, 0.0001, 0.001, 0.01, 0.1, 1, 10, 100 μg/mL) for 24 and 48 h. The CellTiter-Glo assay was performed to measure CM viability. Western blot with grayscale analysis and confocal laser scanning microscopy were used to evaluate the effects of allicin on the synthesis and trafficking of Cav1.2 channel proteins in primary CMs.

**Results:** There was no significant difference in apoptotic toxicity from the actual cell viability (*p* > 0.05) in any group (0, 0.0001, 0.001, 0.01, 0.1, 1, 10, 100 μg/mL allicin), except that viability in the 0.001 and 0.01 μg/mL groups at 24 h were significantly greater (137.37 and 135.96%) (*p* < 0.05). Western blot with grayscale analysis revealed no substantial inhibition by allicin of the synthesis of Cav1.2 proteins. Confocal laser scanning microscopy revealed trafficking dysfunction of Cav1.2 channels caused by allicin in primary CMs.

**Conclusion:** This study is the first to demonstrate that allicin inhibits cardiac Cav1.2 channels by disrupting trafficking, possibly mediating its antiarrhythmic benefits. Therefore, allicin might serve as a new antiarrhythmic agent in the future.

## Introduction

The l-type calcium (Cav1.2) channel is encoded by the CACNA1C gene; it provides a pivotal substrate for cardiac electrophysiologic activities. The Cav1.2 channel conducts L-type calcium current (I_CaL_) that takes part in excitation–contraction (EC) coupling, controlling action potential duration (APD) and regulation of gene expression of cardiac cells, thereby playing an important role in cardiac function (Antzelevitch et al. 2007; Sicouri et al. 2007; Zhao et al. 2012; Navedo & Santana 2013). Mutations in CACNA1C or drug interactions with Cav1.2 channel protein might lead to gain or loss of functions of Cav1.2 channel, giving rise to alterations of I_CaL_ and consequently triggering malignant arrhythmias (Gao et al. 2013; Koenig et al. 2013). With this unique attribute, the Cav1.2 channel may be a primary target for the pharmacological management of arrhythmias.

Garlic and its extract allicin have been increasingly recognized for their effects in the prevention of various ailments. In terms of its pharmacological effects on the cardiovascular system, the correlation between the therapeutic effects of allicin and its side-effects on the heart has attracted widespread attention. To date, allicin has been proved exerting beneficial effects on the progression of subclinical atherosclerosis, regulation of blood lipids, antihypertension, antiplatelet aggregation, balancing blood coagulation and anticoagulation (Bordia et al. 1998; Zahid Ashraf et al. 2005; Allison et al. 2006; Chan et al. 2007).

Recently, studies of the effects of allicin on the cardiac ion channel and APD are underway. Allicin has been shown to reduce the amplitude of I_to_ (transient outward potassium current) and I_Kr_ (the rapid component of the cardiac delayed rectifier potassium current) in a concentration-dependent manner, prolonging APD in cardiomyocytes (CMs) by significantly disrupting the trafficking of corresponding ion channels (Deng et al. 2011; Li G et al. 2014). However, the effects of allicin on Cav1.2 channel remain to be defined. In the present study, we evaluated the effects of allicin on the expression of Cav1.2 channels in primary CMs of neonatal Sprague-Dawley (SD) rats using cytotoxicity assays, western blotting with grayscale analysis and confocal laser scanning microscopy.

## Methods and materials

All animal procedures were conducted in accordance with the principles of the Declaration of Helsinki. The care and use of animals for scientific purposes were approved by the Animal Ethics Committee of Xi’an Jiaotong University.

### Cell culture

A total of 15 male SD rats aged 5–8 weeks and weighing 150–200 g were housed at 25 °C with 30% humidity and a 12 h light/dark cycle, with free access to water and food. At the beginning of the study, the animals were sacrificed by cervical dislocation. The ventricular tissues were cut into sections approximately 1 mm^3^ using ophthalmic scissors, then were washed twice in phosphate buffered saline without calcium or magnesium ions. The tissue pieces were moved into 10 mL centrifuge tubes for further digestion by trypsin and type II collagenase. The CMs were collected by centrifugation (TDZ4-WS, Xiangyi, Shanghai, China) at 500 × *g* for 5 min at room temperature. The isolated cells were cultured at 37 °C in a humidified atmosphere containing 5% CO_2_. The cell culture plates were precoated with 10 μg/mL of fibronectin and the cells were cultured in medium (CM media) containing Dulbecco’s-modified eagle medium (DMEM/F-12) with 2-hydroxyethyl (HEPES) (Invitrogen, Shanghai, China), 10% foetal bovine serum, 1% antibiotic-antimycotic solution (Invitrogen), 3 mM pyruvic acid, 2 mg/mL bovine serum albumin, 100 μg/mL ampicillin and insulin-transferrin-selenium solution (20 ng/mL, 11 ng/mL, and 13.4 pg/mL, respectively; Invitrogen), 5 μg/mL linoleic acid and 100 μM ascorbic acid (Nguyen et al. 2012). For all experiments, cells were cultured at 5 × 10^4^ cells/mL unless otherwise stated.

### Allicin preparation

Allicin was prepared as previously described (Li G et al. 2014). Allicin (74265, Sigma-Aldrich, Merck KGaA, Darmstadt, Germany) was dissolved in dimethyl sulphoxide (DMSO) at a concentration of 10 mg/mL and stored at −4 °C. This stock solution was serially diluted in physiological saline to the final concentrations before each experiment for further application. The final content of DMSO in physiological saline during the experiments did not exceed 0.05%.

### Cell viability determination by CellTiter-Glo assays

CellTiter-Glo is a luminescent assay used to measure cell viability using ATP (adenosine triphosphate) levels. Cells (100 μL, 5 × 10^4^ cells/mL, 5000 cells/well) were continuously incubated with 0, 0.0001, 0.001, 0.01, 0.1, 1, 10, 100 μg/mL allicin, seeded into a 96-well flat-bottomed plates and then incubated for 24 h and 48 h at 37 °C in a humidified atmosphere containing 5% CO_2_ (1 μg/mL allicin ≈ 5.64 µmol/L allicin, the unit of cell viability pre-experiment was μg/mL; therefore, we used μg/mL to normalize the gradients). CellTiter-Glo reagent (50 µL) and 50 µL of DMEM were added to the wells and were then incubated at room temperature for 10 min protected from the light. Luminescence was recorded using Tecan Infinite M1000 multimode plate reader (Tecan Austria GmbH, Grödig, Austria). Values of viability of treated cells were expressed as a percentage of that from corresponding control cells. All experiments were repeated at least three times unless otherwise stated.

### Western blotting with grayscale analysis

Western blotting was conducted according to the manufacturer’s directions. Whole-cell lysates (*n* = 1250 cells, 25 μL, 5 × 10^4^ cells/mL) for each group were prepared with 48 h exposure to 0, 3, 10, 30 µmol/L allicin or vehicle. The choice of the experimental concentrations was based on the results of the cell viability experiment and the results of the previous studies of allicin of our team (Li G et al. 2014). Lysates were centrifuged at 10,000 *g* for 5 min, and protein content was analyzed with BCA Protein Assay Kit (HyClone-Pierce 23225). Five micrograms of protein in each group was separated by running the sample on a NuPage 4–12% Bis-Tris gel (Sangon, Shanghai, China). The proteins were transferred to PVDF membranes (Sangon, 0.45 µm pore) and blocked for 1 h with 10% nonfat dry milk in Tris-buffered saline with Tween 20 (TBST) buffer at 4 °C and then incubated overnight with antibodies targeting CACNA1C (anti-CACNA1C antibody, Abcam, ab58552, 1:400) in TBST with 5% nonfat dry milk at 4 °C. Membranes were washed four times with TBST for 10 min each time and were then incubated with secondary goat anti-rabbit IgG (Santa-Cruz SC-2004 1:3000, California, USA) in TBST buffer with 3% nonfat dry milk at room temperature for 1 h. The blots were then washed four times for 10 min each in TBST, and antibody interactions and the grayscale intensities were measured with the ECL-PLUS/Kit (Amersham, RPN2132).

### Expression and location of Cav1.2 protein with and without allicin

Primary CMs (*n* = 1250 cells, 25 μL, 5 × 10^4^ cells/mL) were cultured in 35 mm glass bottom culture dishes (NEST Biotechnology, Vanguard International Investment Co., Ltd., Hong Kong) and were then continuously incubated with antibodies targeting CACNA1C (Abcam ab58552, Cambridge, UK), Calnexin (Invitrogen MA3-027), Hoechest (Sangon E607329), secondary goat anti-rabbit IgG (Invitrogen 1832035) and goat anti-mouse IgG (Invitrogen 243833) in 0, 3, 10, 30 µmol/L allicin solution for 48 h, respectively. Images of cells were obtained using an FV1000 laser scanning microscope (Leica DM2500, Germany) (Li G et al. 2014).

### Statistical analysis

All values are presented as the means ± SEM. P–P plot and Q–Q plots were used to test for normality. The Levene test was used for homogeneity of variance. Statistical comparisons between two groups were performed by the Dunnett *t*-test and the two-sample *t*-test. All analyses were conducted using SPSS version 22.0 (SPSS Inc., Chicago, IL). Probabilities of *p* < 0.05 were considered statistically significant.

## Results

### Effects of allicin on cell viability by CellTiter-Glo assays

**Figure 1.**
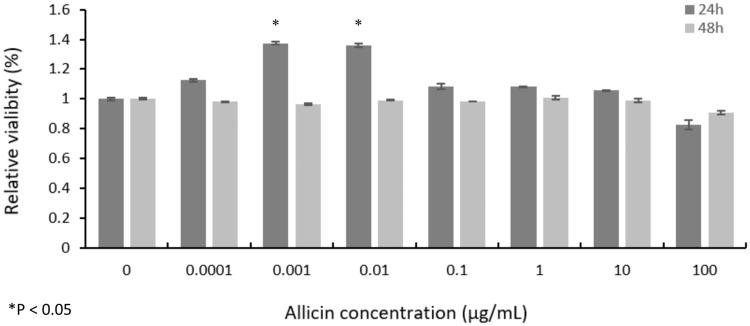
Viability of primary CMs showing no significant difference (*p* > 0.05) at 24 h and 48 h under various allicin concentrations, except that the viability in 0.001 µg/mL and 0.01 µg/mL groups at 24 h significantly increased to 137.37% and 135.96% (*p* < 0.05) (*n* = 5000 cells, 100 μL, 5 × 10^4^ cells/mL).

In the CellTiter-Glo assay, with increased allicin concentrations, there were no significant differences in apoptotic toxicity from the actual cell viability (*p* > 0.05) in any group at 24 and 48 h, except that viabilities in the 0.001 and 0.01 µg/mL groups at 24 h significantly increased to 137.37% (*p* = 0.000003, 95% CI 0.345–0.402) and 135.96% (*p* = 0.000005, 95% CI 0.329–0.390), respectively, compared with the control group (*p* < 0.05) (Figure 1). The concrete mechanism pertaining to the proliferative effect remains to be investigated. In our experiments, the wells without assay reagents were used as blanks and were subtracted from the experimental wells. Furthermore, assay reagents in the absence of cells were also analyzed, and no significant effect on absorbance, fluorescence or luminescent readout was observed. Therefore, we hypothesized that only the increased absorbance and fluorescence (and to a lesser extent, the luminescence) were evident and were elicited by the combination of cells and assay reagents.

### Effects of allicin on Cav1.2 proteins synthesis by Western blot

**Figure 2.**
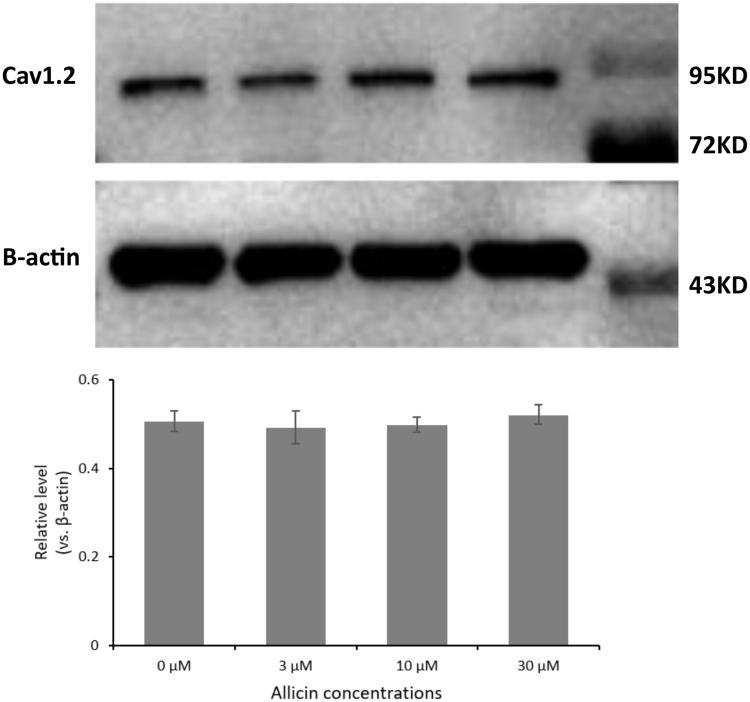
The effect of 48 h allicin exposure on total cellular Cav1.2 proteins. No significant difference was observed in all groups (*p* > 0.05). From left: control, 3 µmol/L allicin, 10 µmol/L allicin, 30 µmol/L allicin, Marker (*n* = 1250 cells, 25 μL, 5 × 10^4^ cells/mL).

According to the results of CellTiter-Glo assay and the previous studies of allicin by our team, three different concentrations of allicin (3, 10 and 30 µmol/L) were used at the end-point of 48 h in subsequent experiments. To ascertain allicin-induced alterations in the synthesis of Cav1.2 proteins, we used Western blot with grayscale analysis to evaluate the effects of allicin on Cav1.2 protein expression in the primary CMs. As shown in Figure 2, no significant difference (*p* > 0.05) was observed between the control group and the 3, 10 and 30 µmol/L allicin groups (*p* = 0.416, 0.458 and 0.206, respectively). We concluded that administration of allicin had no substantial effect on protein synthesis of Cav1.2 channels.

### Effects of allicin on Cav1.2 protein trafficking by confocal laser scanning microscopy

**Figure 3.**
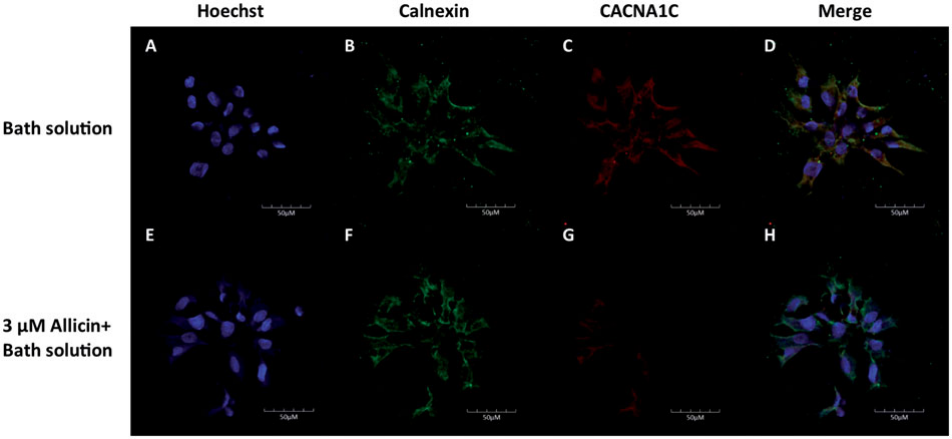
Representative images of subcellular localization of Cav1.2 proteins in primary CMs. The upper line shows representative images of primary CMs without allicin, and the lower row shows representative images of primary CMs with allicin. *n* = 1250 cells, 25 μL, 5 × 10^4^ cells/mL.

To evaluate allicin-induced alterations in the trafficking of Cav1.2 proteins, we used confocal laser scanning microscopy. Images from the control and 3, 10 and 30 µmol/L allicin-incubated cells were compared (Figure 3). Anti-CACNA1C antibody under control conditions was found predominantly on the cell surface, indicating normal trafficking of Cav1.2 proteins from the endoplasmic reticulum to the plasma membrane. However, images of allicin-treated cells in all three groups showed trafficking disruption of Cav1.2 channel proteins from the endoplasmic reticulum to the plasma membrane. Despite the fact that allicin had no impact on the synthesis of Cav1.2 channel proteins, it induced protein trafficking dysfunction. We concluded that the administration of allicin caused Cav1.2 channel protein trafficking abnormalities in primary CMs.

## Discussion

Allicin is the most biologically active substance present in garlic (Chung 2006; Borlinghaus et al. 2014). The antiarrhythmic properties of allicin might result from its blockade of various cardiac ion channels. Cav1.2 channel proteins are extensively distributed and play critical physiological roles in various cells, especially in the heart tissue, contributing to control of APD. To the best of our knowledge, this study is the first to verify the hypothesis that allicin exerts an inhibitory effect on Cav1.2 channels by disrupting trafficking of Cav1.2 channel proteins. This may, at least partly, explain the inhibitory potential of allicin in Cav1.2 channels.

The CellTiter-Glo assay showed that allicin had little apoptotic toxicity on primary CMs. However, the viability in the 0.001 and 0.01 µg/mL allicin groups at 24 h significantly increased to 137.37% and 135.96%, respectively (*p* < 0.05). Allicin has been found to play a similar role in chondrocytes (Li T et al. 2015). By contrast, allicin had antiproliferative effects in HeLa cells (Ilic et al. 2015). To date, the proliferative effect of allicin on CMs has not been reported; therefore, the concrete mechanism remains to be investigated, and this feature might pave the way for CMs culture *in vitro*. We further investigated the influence of allicin on the synthesis of Cav1.2 channel proteins in CMs. Western blot with grayscale analysis revealed that allicin made little impact on the synthesis of Cav1.2 channel proteins, however, confocal laser scanning microscopic images of allicin-treated primary CMs showed a reduction in Cav1.2 channel proteins on the CM membrane and a retention in the cytoplasm. Therefore, allicin had no substantial influence on the Cav1.2 channel proteins synthesis but induced protein trafficking dysfunction in our experiments.

The Cav1.2 channel plays a vital role in the electrophysiologic activities of the heart. It is responsible for EC coupling (Guatimosim et al. 2002), controlling APD, and regulation of gene expression of cardiac cells (Qu et al. 2013). Any slight changes in Ca^2+^ current (I_CaL_) may lead to lethal cardiac events. Therefore, it is reasonable to assume that administration of allicin may have antiarrhythmic effects by its inhibitory potential in Cav1.2 channels as mentioned above.

Specifically, mutations of CACNA1C gene or drug interference with the Cav1.2 channel may generate alterations of its expression and function. Either gain or loss of Cav1.2 functions may result in pro-arrhythmic effects in particular individuals. For example, Timothy syndrome is caused by the G406R mutation in the CACNA1C gene, resulting in a gain of channel function by causing sustained Ca^2+^ influx, promoting APD prolongation and triggering early after-depolarization. These cascade reactions eventually result in torsade de pointes and ventricular fibrillation (Kochhauser et al. 2012; Venetucci et al. 2012; Qu et al. 2013). In theory, allicin serving as an antiarrhythmic agent could participate in pathological courses, down-regulating Cav1.2 channel functions, and preventing malignant arrhythmias.

Although inhibition of Cav1.2 channels is a promising target for antiarrhythmic therapies, this inhibition can also be arrhythmogenic, leading to drug-induced short QT syndrome and Brugada-type ECG (Tse et al. 2017; Turker et al. 2017). With respect to allicin, there are no direct lines of evidence to suggest its arrhythmogenic features. Nevertheless, these pro-arrhythmic characteristics exist theoretically. Consequently, the potential of antiarrhythmic and pro-arrhythmic properties of allicin should be synthetically evaluated during drug development to reduce side-effects.

## Conclusions

This study is the first to demonstrate that allicin inhibits cardiac Cav1.2 channels by disrupting Cav1.2 protein trafficking, possibly explaining its antiarrhythmic benefits. Allicin might serve as a new antiarrhythmic agent in the future.
